# Molecular Toxicity Mechanism Induced by the Antibacterial Agent Triclosan in Freshwater *Euglena gracilis* Based on the Transcriptome

**DOI:** 10.3390/toxics11050414

**Published:** 2023-04-27

**Authors:** Ting Lu, Tong Zhang, Weishu Yang, Bin Yang, Jing Cao, Yang Yang, Mei Li

**Affiliations:** 1 State Key Laboratory of Pollution Control and Resource Reuse, School of Environment, Nanjing University, Nanjing 210023, China; 2 School of Resources and Environmental Sciences, Nanjing Agricultural University, Nanjing 210095, China

**Keywords:** triclosan, *Euglena gracilis*, oxidative stress, photosynthetic pigment, molecular toxicity

## Abstract

Triclosan (TCS), a commonly used antibacterial preservative, has been demonstrated to have high toxicological potential and adversely affects the water bodies. Since algae are one of the most significant primary producers on the planet, understanding the toxicological processes of TCS is critical for determining its risk in aquatic ecosystems and managing the water environment. The physiological and transcriptome changes in *Euglena gracilis* were studied in this study after 7 days of TCS treatment. A distinct inhibition ratio for the photosynthetic pigment content in *E. gracilis* was observed from 2.64% to 37.42% at 0.3–1.2 mg/L, with TCS inhibiting photosynthesis and growth of the algae by up to 38.62%. Superoxide dismutase and glutathione reductase significantly changed after exposure to TCS, compared to the control, indicating that the cellular antioxidant defense responses were induced. Based on transcriptomics, the differentially expressed genes were mainly enriched in biological processes involved in metabolism pathways and microbial metabolism in diverse environments. Integrating transcriptomics and biochemical indicators found that changed reactive oxygen species and antioxidant enzyme activities stimulating algal cell damage and the inhibition of metabolic pathways controlled by the down-regulation of differentially expressed genes were the main toxic mechanisms of TCS exposure to *E. gracilis*. These findings establish the groundwork for future research into the molecular toxicity to microalgae induced by aquatic pollutants, as well as provide fundamental data and recommendations for TCS ecological risk assessment.

## 1. Introduction

Water environmental management issues resulting from the increased wastewater generation associated with antimicrobial agents are a major challenge for countries [1]. Triclosan (TCS), an effective polychlorinated aromatic antibacterial agent, is broadly added to many medical and personal care products to achieve concentrations of 0.1 to 0.3%. In the last few decades, its use has grown exponentially in many products, such as TCS-coated antibacterial sutures, TCS-contained composite materials, hand sanitizer and detergent, and cosmetics [2,3,4]. TCS pollution is widely monitored around the world, due to mass consumption of these and other products. Research shows that TCS continuously enters the aquatic environment, consequently accumulating in water bodies, especially freshwater environments [4,5]. A United States Geological Survey (USGS) report indicates that TCS is one of the top 10 river pollutants in the United States [6]. The global maximum measured TCS concentrations in water are as high as 86 µg/L, 5.3 µg/L, 40µg/L, and 0.1 µg/L for influent, wastewater, surface water, and seawater, respectively [7]. Additionally, the concentration ranges of TCS in the tributary of the Yangtze River, Nanjing, China reached 0.25–0.43 µg/L [8]. More seriously, TCS has been found in urine, breast milk, and blood samples. Specifically, the reported TCS concentration in the urine of Chinese children ranged from none detectable to 681.38 μg/L, and the concentration in blood samples was 0.126–0.161 μg/L [3,8,9]. Considering its potential risk to human health, TCS has been banned in human hygiene products, including household soaps in the United States, since 2017 [10,11]. However, TCS is still widely used in many countries, resulting in high residual levels in aquatic environments, which is potentially harmful for the health of aquatic organisms [4].

As a typical hydrophobic organic compound, TCS exhibits further environmental persistence, based on its high octanol water partition coefficient (log*_Know_* = 4.8) and long half-life [12,13]. Compared to TCS, a variety of conversion products of TCS are more persistent, due to its higher hydrophobicity and lower potential for photodegradation, such as chlorophenols, methyl-triclosan, and dioxins [14]. Research found evidence that an abundance of TCS and its degradation products exist in the environment, especially in the aquatic environment [15,16]. Due to its hydrophobicity, TCS shows high bioaccumulation in organs far exceeding its environmental water concentration [17], which may cause strong toxicity to aquatic organisms, such as algae [4], protozoa [18], insects ([19], crustaceans [20], fish [21,22], and amphibians [23]. Among these aquatic organisms, there are many related studies on the 96 h half-lethal concentration (LC_50_) values of TCS for fish and microalgae. For fish, the LC_50_–96 h was 600.0 μg/L for *Poecilia vivipara* and 1700.0 μg/L for *Oryzias latipes* [24]. For microalgae, TCS induced the median effective concentrations (EC_50_) of 27.1 and 93 μg/L for *Pseudokirchneriella subcapitata* and *Dunaliella tertiolecta,* respectively, while freshwater algae are more sensitive than marine algae and bacteria [25]. Toxicity studies for different algae (excluding *P. subcapitata*) at TCS concentrations ranging from 20.0 to 4000.0 μg/L report that this biocide promotes a reduction in the chlorophyll concentration [26], increases cell membrane activity and permeability [8,27], and interferes with photosynthesis [28]. 

However, most of the current assessments addressing the toxic effects of TCS focus on the typical phenotypic-based endpoints, such as growth, antioxidant activity, and other indicators. Little information is available on the algal responses to TCS on the molecular level [29,30]. Recently, the transcriptomic has often been used to explore the mechanism of toxic effects by detecting the whole gene expression of organisms and providing insight into the cellular biochemistry. Currently, transcriptomic analysis is used to reveal the toxicity mechanisms of nanoparticles, heavy metals, and organic pollutants in algae [31,32,33]. This technique is being used to comprehensively analyze transcriptomic changes and reveal the molecular mechanism caused by TCS toxicity in organisms. Transcriptomics and biochemical research in *Labeo rohita* indicated that TCS caused liver and kidney damage, abnormal metabolic processes, and digestive system disorders [34,35]. Transcriptome analysis of zebrafish revealed the role of the liver as a target organ for TCS toxicity, with liver steatosis mainly resulting from increased fatty acid synthetase activity, and the uptake and suppression of β-oxidation [8]. Moreover, in the green alga *Raphidocelis subcapitata*, TCS suppressed molecular signaling pathways, including porphyrin and chlorophyll metabolism, photosynthesis–antenna proteins, and photosynthesis [36].

*Euglena gracilis*, a secondary green alga that mostly lives in fresh water, has the dual characteristics of flora and fauna [37]. Meanwhile, due to its lack of cell wall and sensitivity to environmental pressure, it is often used as a model organism to evaluate the ecotoxicity of various chemicals [38,39,40]. The purpose of this study was to investigate the harmful effects of various TCS concentrations on *E. gracilis*. The broad transcriptome and metabolic pathway alterations in *E. gracilis* generated by TCS were studied to better understand the underlying toxicological processes. The major genes and molecular pathways reacting to TCS were screened using the enrichment analysis of biological functions and signal pathways. As a result, the alterations in metabolism and gene information processing caused by TCS exposure were explained. These findings contribute to a better understanding of the toxic mechanism of TCS on *E. gracilis* and give new insight into future aquatic environmental toxicological investigations and assessments.

## 2. Materials and Methods

### 2.1. Triclosan and Euglena Gracilis Cultivation

TCS (purity > 97%) was purchased from Aladdin Reagent Co., Ltd. (Shanghai, China). *Euglena gracilis* was obtained from the Freshwater Algae Culture Collection at the Institute of Hydrobiology (FACHB-Collection, Wuhan, China). Checcucci culture medium was used to cultivate the microalgae at 25 ± 1 °C under a 12 h/12 h light/dark cycle, with an illumination intensity of 3000 lux and three replicates [41]. The conical flasks were shaken, and their positions were randomly changed every day to reduce differences in growth among different algal flasks.

### 2.2. Triclosan Exposure

The pretreatment of algae was conducted as per our previous research [39]. The algal inoculum was resuspended 3 days prior to the toxicity tests. The supernatant was removed after centrifugation at 3500× *g* for 15 min. Then, 5 mL of phosphate buffer solution (PBS, Sevier, Wuhan, China) was used to resuspend the algal cells while ensuring that the original microalgal density was in the exponential growth phase at approximately 1 × 10^5^ cells/mL. Briefly, to calculate EC_50_, *E. gracilis* was exposed to different concentrations of TCS (0.3, 0.6, 0.9, and 1.2 mg/L), and blank and acetone solvent control groups (0.15%) were performed. The cell density was measured every 24 h at a wavelength of 680 nm, which had a linear correlation with the cell number, according to our previous study using Multiscan spectroscopy (INFINITE M200, Beijing, China), allowing the establishment of a 96 h growth curve for *E. gracilis* [39]. To further explore the toxicity mechanism, 0.30 mg/L (minimum effective concentration, TCS-E) and 1.20 mg/L (maximum effective concentration, TCS-H) TCS were selected for subsequent transmission electron microscopy (TEM) and transcriptome analysis after 96 h of exposure.

### 2.3. Transmission Electron Microscopy Analysis

After 96 h of exposure, 15 mL of algal solution (>10^7^ cells/mL) from the TCS-E and TCS-H treatments and the acetone solvent control group were centrifuged at 3500× *g* for 15 min in a 1.5 mL centrifuge tube. Subsequently, 1 mL of room temperature 2.5% glutaraldehyde (Aladdin Reagent Co., Ltd., Shanghai, China) was added to the pellet. The cells were fixed at room temperature for 2 h in the dark and then stored at 4 °C prior to analysis. Ultrastructure images were taken by TEM (HITACHI HT7700, Tokyo, Japan).

### 2.4. Pigments Content

After 96 h of exposure to TCS, 1 mL of microalgal suspension from each treatment group was centrifuged at 3500× *g* for 15 min. The supernatant was removed; 1 mL of 80% acetone(Aladdin Reagent Co., Ltd., Shanghai, China) was added to samples and mixed well, and the suspension was then placed at room temperature in the dark for 24 h. After extracting the pigments, the mixture was centrifuged again at 3500× *g* for 15 min, and 80% acetone was used for the control group. The collected supernatant was subjected to Multiscan spectrum analysis at wavelengths of 663, 645, and 470 nm. Chlorophyll a (*Chl a*), chlorophyll *b* (*Chl b*), and carotenoid (*CAR*) contents and the inhibition rate were calculated according to the following equations [42]:

(1) $$Chl\;a=12.21A_{663}-A_{645}$$

(2) $$Chl\;b=20.13A_{645}-5.03A_{663}$$

(3) $$CAR=\left(1000A_{470}-3.27Chl\;a-104Chl\;b\right)/229$$

(4) $$\mathrm{The}\;\mathrm{inhibition}\;\mathrm{rate}=\frac{\mathrm{Sample}\;\mathrm{value}-\mathrm{Control}\;\mathrm{value}}{\mathrm{Control}\;\mathrm{value}}\times 100\%$$

A663, A645, and A470 are the fluorescence values at wavelengths of 663, 645, and 470 nm measured by the microplate reader (Synergy H1, Bio Tek, Winooski, VT, USA).

### 2.5. Oxidative Stress

After 96 h of exposure to TCS, algal cells were centrifuged and rinsed three times with phosphate buffer saline (PBS). The activities of superoxide dismutase (SOD, No. A001-3), glutathione (GSH, No. A006-2), and reactive oxygen species (ROS, No. E004-1) were determined using commercial kits (Jiancheng Bioeng. Inst., Nanjing, China) for the estimation of oxidative stress [43]. The protein content (No. A045-4) was measured by the Coomassie brilliant blue method using a kit (Jiancheng, Nanjing, China) to standardize the enzyme activities. All enzyme activity results were expressed by fluorescence values directly or after calculation via the instructions.

### 2.6. RNA Extraction and Sequencing

After 96 h of exposure to TCS-E and TCS-H, total RNA was extracted from 10 mL of algal cultures (>5 × 10^6^ cells) via TRIzol extraction (Takara, Maebashi, Japan). A NanoDrop instrument was used to determine the RNA concentration, while the RNA integrity was checked by automated electrophoresis on an Agilent 4150 Tapestation system. After meeting the requirements of sequencing and library construction, the library was constructed.

### 2.7. De Novo Transcriptome Assembly

Clean reads were assembled using Trinity software v2.13.2 (Broad Institute and the Hebrew University of Jerusalem, Jerusalem, Israel, 2022), and unigenes were generated by TGICL purification [44]. The assembled unigenes were annotated by comparing the five functional databases, namely the RefSeq nonredundant protein (ftp://ftp.ncbi.nlm.nih.gov/blast/db (accessed on 15 August 2022)) (NR), Pfam (http://pfam.xfam.org/ (accessed on 15 August 2022)), Gene Ontology (GO), Kyoto Encyclopedia of Gene Genotype (KEGG), and SwissProt (http://ftp.ebi.ac.uk/pub/databases/swissprot (accessed on 15 August 2022)) databases. Subsequently, the unigenes were annotated to the corresponding classification by NCBI BLAST 2.6.0 + software [45] to obtain the corresponding functional annotation. GO and KEGG analyses were used to determine the gene function and key biological pathways.

### 2.8. Differential Gene Expression

The FPKM (fragments per kilobase of exon model per million mapped fragments) value of each gene in each sample was calculated by featureCounts software v2.0.3 (Shi Lab, Austin, Australia, 2021) to compare the differential gene expression between samples. Using the read-out data as the input data for horizontal analysis, the differentially expressed genes (DEGs) were analyzed according to DEseq2 (|log_2_foldchange| ≥ 1, *p* value ≤ 0.05). According to the differential gene detection, this experiment classified and enriched the gene ontology (GO) function and analyzed the KEGG biological pathways and enrichment of the obtained DEGs.

### 2.9. Statistical Analysis

Statistical analysis considered the mean value ± standard deviation (mean ± SD) and employed one-way analysis of variance (ANOVA) using SPSS 24.0 software (IBM, Armonk, NY, USA). Statistical differences between treatments were considered significant at *p* < 0.05. The FPKM value of gene expression in the samples was calculated using the featureCounts software, and the DEGs between groups were analyzed using Deseq2 (*p* < 0.05 and |log_2_foldchange| > 1). All samples were performed in triplicate.

## 3. Results

### 3.1. Dose–Effect Relationship between Triclosan and Euglena Gracilis Growth

**Figure 1:**
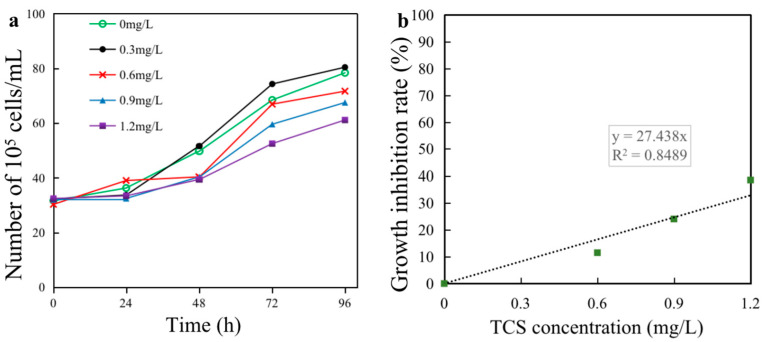
Effect of triclosan (TCS) on the growth of *Euglena gracilis*. (**a**) Cell density; (**b**) growth inhibition rate.

The effects on *E. gracilis* growth were explored under exposure to different concentrations of TCS (0, 0.3, 0.6, 0.9, and 1.2 mg/L). During the 96 h exposure, the *E. gracilis* cell density began to decline, compared with the controls (Figure 1a). With prolonged TCS exposure, the algal cell density in all treatments showed an upwards trend within 24 h, except for the highest concentration group. After 96 h of exposure, a dramatic decrease in the algal growth rate was observed with the incremental TCS concentrations. The growth inhibition rate of algae exposed to 0.6, 0.9, and 1.2 mg/L TCS for 96 h decreased to 11.52%, 24.11%, and 38.62%, respectively, of the control (Figure 1b). The higher the TCS concentration, the more severe the *E. gracilis* growth inhibition, forming a significant dose–effect relationship (Figure 1b). According to the linear interpolation, the 96 h EC_50_ value was calculated to be 1.82 mg/L. The acute toxicity to *E. gracilis* due to TCS exposure was moderately toxic (0.3 mg/L < EC_50_ ≤ 3.0 mg/L), according to the toxicity classification of aquatic organisms (GB/T 31270.14-2014).

### 3.2. Effects on Cell Morphology and Ultrastructure

**Figure 2:**
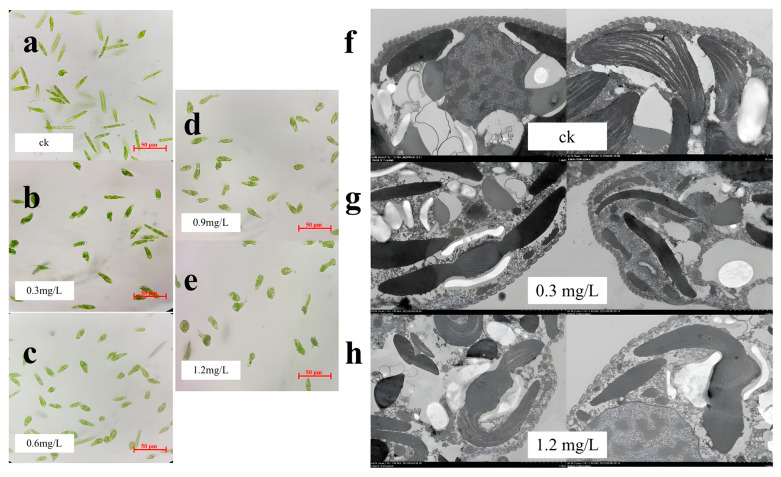
Observations of algal cell morphology. Optical microscope image of the control group (**a**), 0.3 mg/L (**b**), 0.6 mg/L (**c**), 0.9 mg/L (**d**), and 1.2 mg/L (**e**) TCS: the morphology changes in *E. gracilis*; TEM images of the control group (**f**), 0.3 mg/L (**g**), and 1.2 mg/L (**h**) TCS: the ultrastructure of *E. gracilis*, such as the number of vacuoles and chloroplast arrangement and damage.

After 96 h of exposure, the morphology of the algae was observed under a 40× optical microscope. The results implied that the algae gradually tended to become distorted, and there was an increasing number of deformed algae with an increasing TCS concentration (Figure 2a–e). All *E. gracilis* cells after exposure to TCS had morphological deformities. TEM images were used to observe the microalgal ultrastructure after TCS exposure for 96 h (Figure 2f–h). After exposure to TCS, the number of vacuoles in the *E. gracilis* cells increased notably. Morphological changes in the algal cells were observed after treatment with TCS, including the fragmentation of cells around the chloroplasts. Compared with the control, the chloroplast membranes of *E. gracilis* cells exposed to 0.3 and 1.2 mg/L TCS were slightly abnormal and loosely arranged, which was consistent with the optical microscopy observations. This implies that the growth of *E. gracilis* may be inhibited through toxicity caused by the exposure of chloroplasts to TCS. 

### 3.3. Physiological Index Changes Induced by Triclosan Exposure

**Figure 3:**
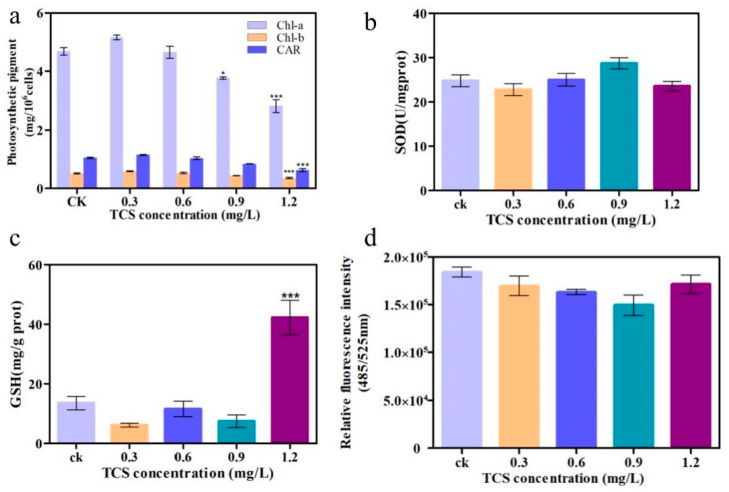
Changes in physiological indicators induced by triclosan (TCS). (**a**) Photosynthetic pigment content; (**b**) the activities of superoxide dismutase (SOD); (**c**) the activities of glutathione (GSH); and (**d**) the activities of relative fluorescence intensity. * *p* < 0.05, *** *p* < 0.001, compared with the control group.

The photosynthetic pigment content is an important toxicological index of algae and is widely used to indicate the effects of pollutants on photosynthesis. For *E. gracilis*, all photosynthetic pigment indices (Chl-*a*, Chl-*b* and *CAR*) significantly decreased at high TCS concentrations (1.2 mg/L), compared to the control (*p* < 0.01) (Figure 3a), and the inhibition rates reached 37.42%, 32.23%, and 35.80%, respectively, for Chl-*a*, Chl-*b,* and *CAR*. Subsequently, the activities of SOD and GSH, which are essential enzymes of the antioxidative system, were determined. TCS generally changed the activities of SOD and GSH in a concentration-independent manner. SOD activity was inhibited by 24% at 1.2 mg/L TCS (Figure 3b). In contrast, the activities of the GSH level were significantly higher than those of the control (Figure 3c), reaching 303.47%, which indicates that oxidative damage occurred in the algae exposed to a high TCS concentration. Meanwhile, the ROS level declined with the increasing concentration of TCS, although it increased at 1.2 mg/L (Figure 3d). Thus, 1.2 mg/L was considered the critical TCS concentration for algae, after which the ROS levels might exceed its own regulation/detoxification ability. These findings show that exposure to high concentrations of TCS (1.2 mg/L) can induce oxidative stress and reduce photosynthetic pigment in the microalgae, leading to an increase in GSH and ROS production and a decrease in SOD activity. 

### 3.4. Transcriptome Analysis

**Figure 4:**
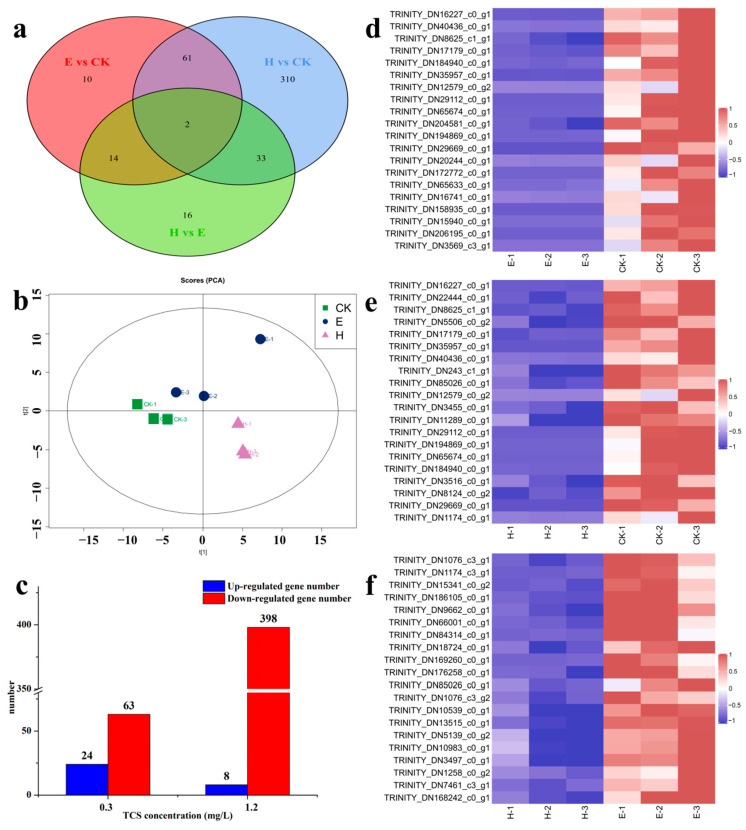
(**a**) Venn diagram of differentially expressed genes (DEGs); (**b**) principal component analysis (PCA) for *Euglena gracilis*; (**c**) differentially expressed gene (DEG) numbers due to triclosan (TCS) exposure (FDR < 0.05); clustering heat map of the top 20 DEGs between groups; (**d**) TCS-E (0.3 mg/L) vs. control; (**e**) TCS-H (1.2 mg/L) vs. control; and (**f**) TCS-H (1.2 mg/L) vs. TCS-E (0.3 mg/L).

To explore the molecular toxicity mechanism of TCS to *E. gracilis*, TCS-E and TCS-H (Figure 3) were selected for transcriptomics analysis, according to their toxic effects. A total of 285,020 unigenes were obtained from the *E. gracilis* cells. Among the Venn diagrams (Figure 4a), 63 DEGs were shared among all TCS treatments. The histogram showed 87 and 406 DEGs in the TCS-E and TCS-H exposures, respectively, compared to the control (Figure 4c), suggesting that TCS-H induced more severe transcriptional changes. These results were further supported by the principal component analysis (PCA) loading plot (Figure 4b), which exhibited a clear separation between the control and contaminant treatments, especially for the TCS-H exposure. Furthermore, the hierarchical clustering heat map showing the abundances of the top 20 DEGs for TCS-E and TCS-H exhibited downward response trends, with 11 shared DEGs (Figure 4d,e), indicating that the transcriptome response patterns were similar. Compared to TCS-E, there were 65 DEGs in TCS-H, with the top 20 DEGs significantly decreased (Figure 4f). The adverse effect of TCS-H exposure on *E. gracilis* might be greater from the perspective of the transcriptome, and TCS exposure mainly interfered with its growth viability by inhibiting gene expression. 

**Figure 5:**
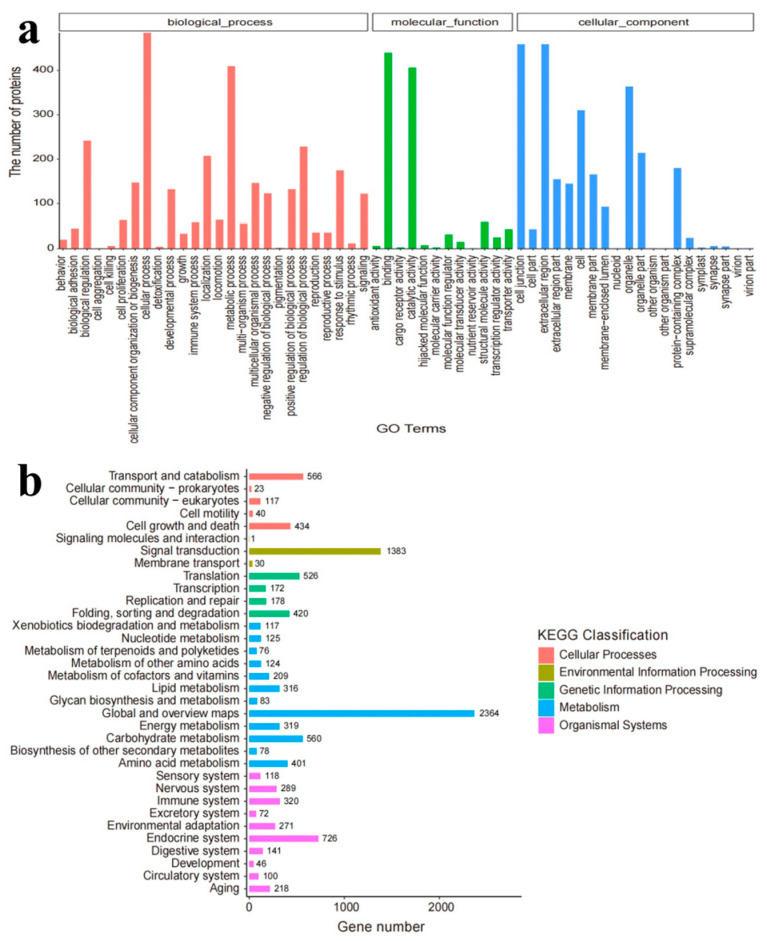
(**a**) Gene classification of *E. gracilis* according to the GO database; (**b**) gene pathway classification of *E. gracilis* according to the KEGG database.

According to the GO functional analysis, biological processes were the most abundant functional gene-encoding products, followed by cellular components and molecular functions (Figure 5a). Cellular and metabolic processes were significantly affected within the biological processes. A total of 10,963 unigenes were annotated into 128 KEGG pathways. Among them, the most significant 34 pathways were enriched (Figure 5b) and divided into 5 KEGG classifications, including metabolism (43.53%) and organic systems (20.99%). Thus, TCS exposure mainly altered the metabolic processes of *E. gracilis.*

**Figure 6:**
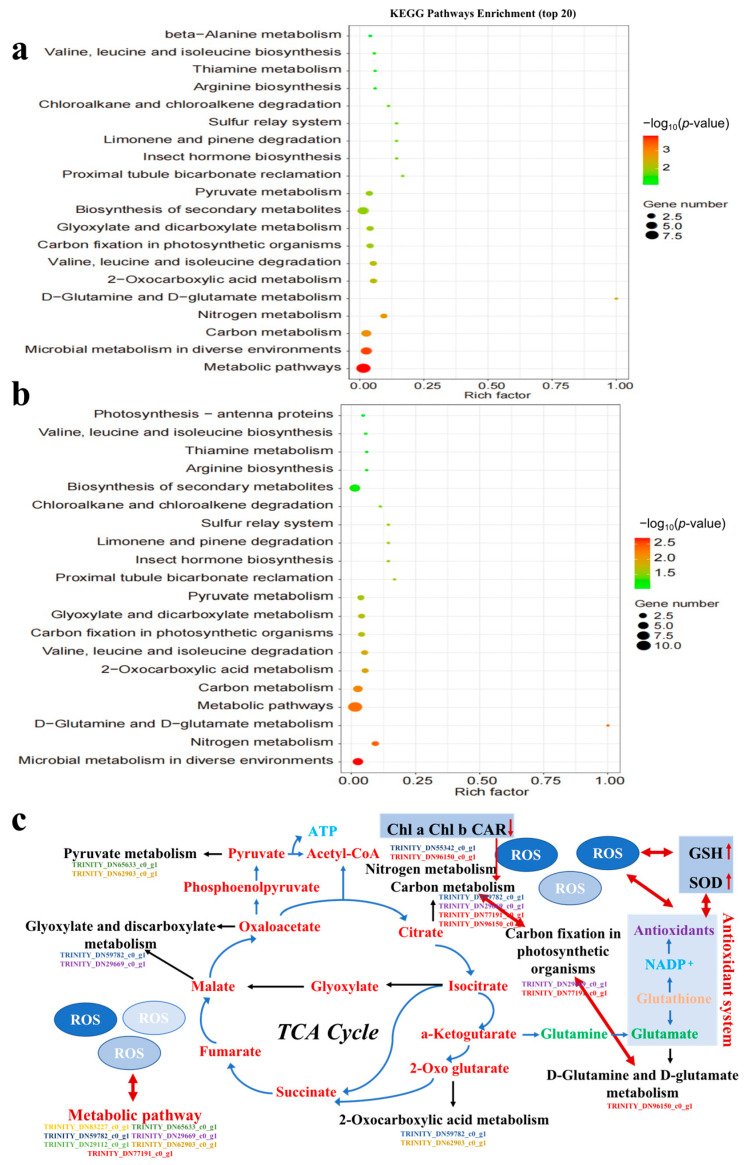
KEGG pathway analysis of *Euglena gracilis* induced by triclosan (TCS). (**a**) TCS-E; (**b**) TCS-H; (**c**) schematic of the proposed metabolic pathways of *Euglena gracilis*.

The top 20 pathways were selected by functional analysis of DEGs in the secondary KEGG pathways (Figure 6a,b), with 19 pathways shared between TCS-E and TCS-H. Almost all significantly altered pathways were shared, such as metabolic pathways, microbial metabolism in diverse environments, carbon metabolism, and biosynthesis of secondary metabolites. This demonstrates the similar toxic mechanism over the range of TCS concentrations (0.3–1.2 mg/L), while TCS-H had more DEGs in the KEGG-enriched pathways, which indicates its greater toxicity. Furthermore, the KEGG pathway showed that carbon and nitrogen metabolism in algae were significantly altered, which is important for the growth and development of *E. gracilis*, especially the carbon fixation of this photosynthetic organism [46]. 

As shown in Figure 6c, the tricarboxylic acid (TCA) cycle is a pathway related to energy and metabolism, and DEGs cause damage to the related pathway and affect the up- and down-regulations of metabolites, ultimately resulting in metabolization-related toxicity to algae. All DEGs were down-regulated in the KEGG-enriched pathways of the glyoxylate and dicarboxylate metabolisms; pyruvate metabolism; carbon fixation in photosynthetic organisms; 2-oxocarboxylic acid metabolism; D-glutamine and D-glutamate metabolism; and nitrogen metabolism. The metabolic and carbon metabolism pathways, representing 7 and 4 DEGs, respectively, exhibited more disturbances than other pathways. Each DEG could control multiple metabolic pathways, indicating that these metabolic pathways were interrelated. For example, carbon fixation in photosynthetic organisms and carbon metabolism shared two DEGs (*TRINITY_DN29669_c0_g1*, *TRINITY_DN77191_c0_g1*) that were significantly down-regulated, resulting in the significant inhibition of photosynthesis in *E. gracilis*, and all the other enrichment pathways shared genes with metabolic pathways.

## 4. Discussion

In order to explore the toxic effect and mechanism of TCS on *E. gracilis*, an acute toxicity test was carried out. The results showed that TCS concentration formed a significant dose–effect relationship with the growth inhibition rate of *E. gracilis* (Figure 1b), which is consistent with the study of *Chlamydomonas reinhardtii* [26]. The sensitivity of different types of algae to TCS was variable. Compared with the EC_50_–96 h values of the green algae *Microcystis aeruginosa* (9.2 μg/L) and *Scenedesmus* subspicatus (2.8 μg/L) and the EC_50_–72 h value of the diatom *Navicula* sp. (145.6 μg/L), *E. gracilis* (1820 μg/L) displayed stronger tolerance to TCS. This phenomenon may be related to the nutrients, water quality, and algal morphology required for the growth of the different algae. In addition, as a heterotroph, *E. gracilis* can survive in the dark, indicating that it may still survive after chloroplast damage, which may be the reason for the high resistance of *E. gracilis* to TCS, compared with other algae [47].

Electron microscope and TEM images were used to observe the microalgal cell damage after TCS exposure for 96 h (Figure 2a–h). In the results, an increased TCS dose induced the morphology of the algae to be more spherical, rather than a slender strip, due to enhanced osmotic stress, indicating algal adaptation to external pressure [48,49]. All *E. gracilis* cells after exposure to TCS had morphological deformities, which may be related to the occurrence of cell pellicule rupture and degradation [50]. After exposure to TCS, the number of vacuoles in the *E. gracilis* cells increased notably, indicating that toxic substances in the algal cell may be transferred into the vacuoles, where antioxidant molecules, such as glutathione S-transferase (GST), can protect or detoxify the algae [51]. Compared with the control, the chloroplast membranes of *E. gracilis* cells exposed to 0.3 and 1.2 mg/L TCS were slightly abnormal and loosely arranged, indicating that the growth of *E. gracilis* may be inhibited through toxicity caused by the exposure of chloroplasts to TCS. 

Photosynthetic pigment content is an important toxicological test index of algae and is widely used to indicate the effect of pollutants on photosynthesis [42]. As shown in Figure 3a, a decline in the algal photosynthetic pigment content after exposure to TCS caused a reduction in photosynthetic activity. Meanwhile, research shows that the exposure of *P. subcapitata* to TCS also induced a reduction in photosynthetic pigments *Chl a*, *Chl b*, and photosynthetic activity [4]. Combined with the conclusions in Figure 2, it was shown that TCS exposure caused cell damage and chloroplast damage in *E. gracilis*, resulting in the hindrance of chlorophyll synthesis, which is the main reason for the reduction in photosynthetic pigments and the inhibition of photosynthesis. 

Furthermore, the oxidative stress response of *E. gracilis* under TCS stress was studied to further understand its tolerance and adaptability to TCS stress. The activities of SOD and GSH, which are essential enzymes of the antioxidative system, were determined. An increase in SOD levels and an increase in GSH levels were caused by the elimination of excess oxygen free radicals and hydrogen peroxide in cells [52]. Meanwhile, the ROS level increased at 1.2 mg/L (Figure 3d). To avoid oxidative damage caused by excessive ROS, the balance between the production and elimination of ROS by enzymatic antioxidants is critical for microalgae [53]. Moreover, lipid peroxidation and membrane structure damage of algal cells exposed to high concentrations of TCS may be one of the explanations for the hindrance of chlorophyll synthesis, which results in cell photosynthesis and growth inhibition. Dioxins, one of the transformation products of TCS, significantly enhances the oxidative stress response of organisms, thereby causing irreversible oxidative damage to organisms, indicating that dioxins produced by TCS transformation may also cause oxidative damage in *E. gracilis* [54]. In conclusion, the exposure of TCS and its transformation products resulted in oxidative stress, triggering cell structure damage and the impairment of cell function in *E. gracilis*, which can further threaten the growth and reproduction of the population. 

From the perspective of the transcriptome, the higher number of DEGs from TCS-H exposure suggested that the adverse effect of high TCS exposure on *E. gracilis* might be greater (Figure 4a), indicating that TCS-H induced more severe transcriptional changes. To reflect an in-depth understanding of the toxicity mechanism, analyses were performed on the GO- and KEGG-enriched pathways. The enrichment results of GO verified that metabolic processes were the most affected, which are closely related to the growth, development, and reproduction of the population. In addition, there were enriched pathways related to oxidative stress. This is because ROS, such as superoxide (O^2−^), hydroxyl radicals (OH^•^), and hydrogen peroxide (H_2_O_2_), are generated in cells when exposed to chemical stresses, such as metals, nanomaterials, and organic chemicals [51]. This result suggests that TCS exposure caused serious damage to the antioxidant system of *E. gracilis*, which is related to the changes in the antioxidant enzyme activity, and in addition, ROS production stimulated enriched pathways related to oxidative stress, which is the main mechanism of the toxic effects of TCS exposure in *E. gracilis*. In addition, through KEGG secondary pathway function and enrichment analysis, it was shown that both TCS-E and TCS-H exposure affected genes related to *E. gracilis* metabolism, suggesting that the metabolic mechanism of algae was blocked under greater toxicity, resulting in a corresponding stress response. This result was consistent with Liao et al. (2020) [42], who found that the combined exposure group of cadmium and microplastics mainly enriched the DEGs of *E. gracilis* in the gene pathways related to metabolism, suggesting that the metabolic mechanism of algae was blocked under the action of greater toxicity, resulting in a corresponding stress response. Studies have shown that organochlorines can induce inflammatory processes in organisms, stimulate oxidative stress responses, and have a bidirectional relationship with endocrine disorders, eventually leading to a variety of metabolic diseases [55]. As a ubiquitous organochlorine, TCS also has the potential to cause metabolic disorders. The KEGG pathway showed that highly coordinated carbon and nitrogen metabolism in the unicellular algae was significantly altered and resulted in the significant inhibition of algal photosynthesis [46]. A greater carbon source was required for nitrogen metabolism in the TCS-H group to enhance the D-glutamine and D-glutamate metabolism cycles, which may be the main reason for the difference in the carbon fixation pathway, compared to TCS-E. Herein, TCS exposure may further affect metabolic pathways by stimulating oxidative stress responses, thereby inhibiting the growth and development of *E. gracilis*, especially the inhibition of photosynthesis by carbon and nitrogen metabolism.

The TCA cycle is a central pathway of primary metabolism for energy production [56], as shown in Figure 6c; it is difficult to speculate which metabolic processes are most influenced by it. The TCA cycle likely serves to make carbon available from amino acids, fatty acids, and other carbon-containing molecules for energy generation [57]. When genes involved in the TCA cycle are suppressed, a reduction in energy-consuming sugar biosynthesis is expected, resulting in photosynthesis inhibition. Meanwhile, algal cells exposed to pollutants require more essential nutrients to survive because the pollutants can accumulate in the algal cell, impeding the absorption/uptake of essential nutrients, thereby inhibiting growth. When exposed to environmental pollutants, the balance between endogenous and exogenous ROS and antioxidant enzyme activity in organisms may be interrupted, and the change in them can subsequently result in oxidative damage to and metabolic disorders in organisms [58]. In Figure 6c, ROS production damaged the antioxidant system; it caused the change in the metabolite glutamate, affected the D-glutamine and D-glutamate metabolism pathways, and down-regulated related the genes in *TRINITY_DN96150_c0_g1* existing in multiple pathways (ko00220, ko00250, ko00471, and so on), which could lead to the disorder of the TCA cycle metabolic system, including carbon and nitrogen metabolism. Herein, the down-regulation of metabolic pathways, as indicated by DEGs, and the change in ROS and the antioxidant enzyme activity mainly caused oxidative stress and photosynthesis inhibition in *E. gracilis*.

Based on the observed responses of physiological biomarkers and transcriptomic analysis, *E. gracilis* exposed to TCS exhibited an inhibition of population growth, with oxidative damage and metabolic pathways significantly altered. As the primary producer, the pollutants consumed by microalgae are easily transferred to larger organisms along the food chain, such as zooplankton, and is potentially harmful to the entire ecological environment and humans [59]. The inhibition of microalgal population growth indicates an increasing ecological risk to other aquatic species, which may lead to a population decline for higher organisms. Furthermore, TCS has a high adsorption potential, allowing it to adsorb to sedimented sewage sludge and migrate to the soil environment [12], which may cause similar ecological risks to the soil environment, thereby affecting soil microorganisms, plants, and animals. Therefore, exploring the toxic mechanism of TCS on *E. gracilis* not only brings more attention to the harm of pollutants in daily necessities, it also lays the foundation for adequate water environment management and studying the toxic effects of pollutants on other aquatic and terrestrial organisms.

## 5. Conclusions

In this study, the adverse effects of TCS on freshwater microalgae (*E. gracilis*), including morphological alterations, reduced photosynthesis, and oxidative stress, were investigated. Additionally, the cell’s own capacity for detoxification was surpassed by the ongoing stress of greater TCS concentrations (1.2 mg/L). It was shown that TCS, to some extent, interfered with the metabolism and gene information processing of *E. gracilis*, leading to neuronal death brought on by oxidative stress damage from functional analysis of DEGs utilizing the GO and KEGG pathways. Therefore, the main toxic mechanisms of TCS exposure to *E. gracilis* were the changes in ROS and antioxidant enzyme activities to stimulate algal cell damage and the inhibition of the TCA cycle metabolic system, including carbon metabolism, nitrogen metabolism, and D-glutamine and D-glutamate metabolism pathways controlled by the down-regulation of DEGs, which were further manifested as oxidative stress and photosynthesis inhibition effects. These results serve as a starting point for further investigation into the specific molecular pathways in microalgae that are affected negatively by the toxic effects of aquatic pollutants. At the same time, it provides theoretical guidance for the application of antibacterial agents in aquatic environments and promotes water environment management.

## Data Availability

Not applicable.
